# The Effect of Triploidy on Gonadal Development, Hematology and Biochemistry in the European Grayling (*Thymallus thymallus*)

**DOI:** 10.3390/ani15040481

**Published:** 2025-02-08

**Authors:** Rafał Rożyński, Stefan Dobosz, Maciej Rożyński, Konrad Ocalewicz

**Affiliations:** 1 Department of Marine Biology and Biotechnology, Faculty of Oceanography and Geography, University of Gdansk, 81-378 Gdynia, Poland; konrad.ocalewicz@ug.edu.pl; 2 Department of Salmonid Research, Inland Fisheries Institute in Olsztyn, 83-330 Żukowo, Poland; s.dobosz@infish.com.pl; 3 Department of Aquaculture, Inland Fisheries Institute in Olsztyn, 10-719 Olsztyn, Poland; m.rozynski@infish.com.pl

**Keywords:** biochemical indices, European grayling, genetic introgression, hematology, protecting native populations, triploidization, sterility, stocking

## Abstract

Sterile triploid European graylings (*Thymallus thymallus*) could serve as an alternative to allochthonous stocking, potentially protecting native populations from genetic introgression. In this study, two-year-old triploid and diploid graylings were examined to assess their hematological and biochemical characteristics and to evaluate the development of their gonads. When compared to diploids, triploids exhibited elevated white blood cell counts, mean corpuscular hemoglobin (MCH) and mean corpuscular hemoglobin concentration (MCHC) by 5.66%, 162.68% and 207.57%, respectively. Moreover, the diameters of red blood cells and their nuclei were significantly higher in triploid graylings. In contrast, the red blood cell count, hemoglobin concentration (Hb), hematocrit and mean corpuscular volume (MCV) were lower in triploids by 64.82%, 5.80%, 70.16% and 14.49%, respectively. Most blood plasma biochemical indices showed no significant differences between specimens of different ploidies; however, triploids had a 21.96% higher level of triglycerides, while diploids had 3.74% more albumin. Additionally, the chloride concentration was 4.74% lower in triploids. Examined diploid males exhibited well-developed, sometimes asymmetrical testes and were actively spermiating. Triploid males were non-spermiating, and their testes were solid with varying morphology. Ovaries in diploid females contained mature oocytes, and in about 30% of the females, the body cavities were filled with ovulated eggs. In turn, the ovaries in triploid females were significantly reduced, usually transparent and lacked any oocytes. However, a longer study over a period exceeding two years needs to be performed to state unequivocally that triploid grayling females are sterile.

## Simple Summary

Two-year-old triploid European graylings (*Thymmallus thymallus*) produced by the application of the high hydrostatic pressure (HHP) shock exhibited an increased white blood cell count (WBC), mean corpuscular hemoglobin (MCH) and mean corpuscular hemoglobin concentration (MCHC) when compared to their diploid siblings. On the other hand, the red blood cell count (RBC), hemoglobin concentration (Hb), hematocrit and mean corpuscular volume (MCV) were significantly lower in the triploid individuals. While diploid graylings had well-developed gonads filled with gametes, triploids were found to have significant problems with gonadal differentiation; ovaries were reduced without any oocytes, and testes were solid and non-spermiating.

## 1. Introduction

The presence of three sets of chromosomes in somatic cells is defined as triploidy [1]. Spontaneous triploid fish arise during meiotic alterations within a species (autotriploids) and in interspecies hybrids (allotriploids) [1]. Disturbances in the gametogenesis process, caused by cytogenetic changes during meiosis including the pre-meiotic endoduplication of the chromosome set and the suppression of the first or second meiotic division [2], result in the development of autotriploids and have been observed in several fish species, including California roach (*Hesperoleucus symmetricus* Baird & Girard, 1854) [3], rainbow trout (*Oncorhynchus mykiss*) [4,5], nurse shark (*Ginglymostoma cirratum* Bonnaterre, 1788) [6] and pond loach (*Misgus anguillicaudatus* Cantor, 1842) [7], among others. An increased ratio of triploids is observed among fish embryos developing in post-ovulatory (aged) eggs, which are characterized by cytoskeletal alterations that lead to the suppression of the second meiotic division [8,9]. In contrast, allotriploids possess three sets of chromosomes derived from different species [1]. Such situations have been described, among others, in hybrid complexes of *Cobitis* (loaches) [10,11,12,13], Prussian carp (*Carassius gibelio* Bloch, 1782) [10,13], hybrid minnow *Squalius alburnoides* [14] and pond loach [7,15]. In these cases, triploid females are fertile and can reproduce gynogenetically, producing non-reduced triploid eggs [1]. Triploid fish can also be generated intentionally by mating diploid and tetraploid individuals [10,16,17,18,19,20] or by the dispermic fertilization of a haploid egg [21,22]. Furthermore, chemical or physical shock applied to fertilized eggs disrupts the microtubules of the meiotic spindle, preventing the extrusion of the second polar body and resulting in the development of triploid embryos [23,24]. The additional set of chromosomes in triploid specimens causes cytogenetic incompatibility that impairs proper gonadal development and gamete production [25]. Triploid females usually exhibit highly reduced ovaries that are unable to produce eggs. In contrast, triploid males develop testes comparable in size to those observed in diploid individuals; however, such fish are generally infertile due to the aneuploidy of their spermatozoa [1,25]. Triploid fish do not experience a decline in growth, survival or meat quality, which typically accompanies sexual maturation in normal diploid specimens. Thus, artificial triploidization is a promising method for cultivating fish species that exhibit early sexual maturation [1,26]. Moreover, the sterility of triploid individuals inhibits interactions between fish from wild stocks and those that have escaped from fish farms or are deliberately released into rivers or lakes as part of stocking programs for recreational fishing purposes.

The European grayling (*Thymallus thymallus* L.) is considered one of the most attractive species for fly fishing in Europe; however, it is also one of the salmonids that is endangered or even critically endangered [27,28,29,30,31]. The distribution range and size of specific populations of the European grayling have decreased due to water pollution, habitat destruction, river engineering, predation by birds and intensive fishing [27,28,29,30,31]. Unfortunately, restocking with farmed specimens has resulted in the contamination of many local European grayling populations with non-native individuals [32,33]. To produce safe stocking material, a protocol for the production of triploid graylings has recently been developed [34].

European graylings mature sexually in the second year of life [5]. The only available data on gonadal development in triploid graylings were collected from much younger specimens; gonads of one-year-old diploid and triploid graylings were macroscopically similar in most of the examined individuals, and only histological analysis confirmed that triploids had problems with gonadal development [34]. As only sterile triploids may be considered safe stocking material, the main purpose of the present research was to evaluate gonadal development in sexually matured two-year-old diploid graylings and their triploid siblings. We also took advantage of the fact that the sampled individuals were of a size enabling blood collection and estimated hematological and plasma biochemical indices that may help to monitor fish health and physiological status [35,36,37]. The hematology examination and assessment of welfare-related blood parameters (concentration of glucose and triglycerides, among others) are particularly important in triploids as fishes with an additional set of chromosomes usually have higher environmental requirements than diploids and are less tolerant than diploids to nonoptimal rearing conditions [1].

## 2. Materials and Methods

### 2.1. Fish, Origin and Rearing Conditions

Diploid (*n* = 40) and triploid (*n* = 40) European graylings from the families established and kept in the Department of Salmonid Research (DSR) of the Inland Fisheries Institute (IFI) in Olsztyn, Rutki, Poland, were sampled (13–14 June 2022) and studied. Triploid families of the European grayling were generated by the application of a recently elaborated protocol that involves the application of a 5 min high hydrostatic pressure (HHP) shock (9000 psi) 20 min after egg insemination using a TRC-APV electric/hydraulic device (TRC Hydraulics Inc. in Dieppe, NB, Canada) [34]. Examined individuals were the offspring of five females, and eight diploid and eight triploid siblings from each family were randomly selected for the experiment. The body length and body weight of diploids ranged from 22.7 to 27.7 cm [(25.1 ± 1.2 cm (mean ± SD)] and from 87.3 to 162.2 g [(118.4 ± 15.9 g (mean ± SD)], respectively. In the case of triploids, body length varied from 23.0 to 28.7 cm (25.8 ± 1.5 cm) and body weight varied from 91.7 to 174.2 g (124.5 ± 22.6 g). Both diploid and triploid grayling individuals were reared separately under the same husbandry conditions in hatchery plastic tanks of 0.3 m^3^ (first year) and 1.2 m^3^ (second year) in size. The fish were fed daily, and feeding rates were adjusted to their growth and diurnal temperature. European graylings from both stocks were fed with the feed formulated for rainbow trout. For the first month, the fish were fed with Aller Infa EX GR 0.4 mm granulated feed and then Aller Futura EX GR 0.5–1.0 mm and Aller Futura EX GR 0.9–1.6 mm. Juvenile and adult individuals were fed with Aller Futura EX GR 1.3–2.0 mm pellets (Aller Aqua A/S, Christiansfeld, Denmark) containing the following: 58% crude protein, 17% crude fat, 6.1% nitrogen-free extract (NFE), 12.2% ash and 0.7% fiber, with a total energy of 21.6 MJ kg^−1^ of feed (manufacturer data).

### 2.2. Blood Sampling

Prior to blood collection, the fish were anesthetized using an aqueous solution of MS-222 at a dose of 100 mg L^−1^ buffered with sodium bicarbonate (2 min, Sigma-Aldrich, St. Louis, MO, USA) [34,38]. Blood was collected with heparinized syringes (Smiths Medical International ASD, Inc., St. Paul, MN, USA) directly from the caudal vein (approximately 1 mL of blood from each individual). Immediately after blood collection, the fish were euthanized without regaining consciousness using an aqueous solution of MS-222 at a dose of 200 mg L^−1^. Preparations for the erythrocyte measurements were made by the smearing of the heparinized whole blood on the microscopic slides. The preparations of blood were air-dried, then fixed in methanol for 20 min and finally stained with 10% Giemsa solution for 15 min.

### 2.3. The Macroscopic Morphology of the Gonads

After the blood collection procedures, the euthanized specimens were subjected to a macroscopic analysis of the gonads. For this purpose, the abdominal integuments of each individual were cut open, and the gonads were observed to determine their morphology.

The testes of each male were assigned to one of the following categories:

(A) Milky white, symmetrical, spermiating;

(B) Milky white, symmetrical, non-spermiating;

(C) Milky white, asymmetrical or segmented, spermiating;

(D) Milky white, asymmetrical or segmented, non-spermiating;

(E) Pinkish, symmetrical, non-spermiating;

(F) Pinkish, asymmetrical or segmented, non-spermiating.

The ovaries were assigned to the following categories:

(A) Yellow–orange, symmetrical with observed non-ovulated oocytes;

(B) Yellow–orange, symmetrical with ovulated oocytes;

(C) Yellow–orange, asymmetrical with observed non-ovulated oocytes;

(D) Yellow–orange, asymmetrical with ovulated oocytes;

(E) Pinkish milky, reduced, symmetrical, no oocytes;

(F) Pinkish transparent, reduced, symmetrical, no oocytes;

(G) Pinkish transparent, reduced, asymmetrical, no oocytes.

### 2.4. Erythrocyte Measurements

The cell (C) and nucleus (N) diameters of fifty erythrocytes from each sampled specimen were measured using the microscope Eclipse E600 (NIKON, Tokio, Japan) equipped with the camera and software NIS-Elements BR 3.2 (NIKON, Tokio, Japan). Then, the nucleocytoplasmic index (NCI) was calculated (NCI = N × C^−1^) [39].

### 2.5. Automatic Measurement of Hematological and Biochemical Indices

Blood samples (approximately 1 mL) were collected from the caudal vein of the anesthetized individuals using heparinized syringes (Smiths Medical International ASD, Inc., St. Paul, MN, USA). Hematological parameters including white blood cell count (WBC), red blood cell count (RBC), hemoglobin (HGB), hematocrit (HCT), mean corpuscular volume (MCV), mean corpuscular hemoglobin (MCH) and mean corpuscular hemoglobin concentration (MCHC) were determined. Biochemical indices including glucose (GLU), triglycerides (TGs), cholesterol (CHOL), total protein (TP), albumin (ALB), globulin (GLB), total bilirubin (BIL-T), creatinine (KREA), lactate (LACT), ammonia (AMON), alanine transaminase (ALT), aspartate transaminase (AST), alkaline phosphatase (ALP), amylase (AMYL), potassium (K^+^), sodium (Na^+^), chloride (Cl^−^), phosphorus (P), calcium (Ca) and magnesium (Mg^2+^) ions were measured in the blood plasma provided by the centrifugation of the blood at 4000 rpm for 3 min (Fresco 17, Thermo Scientific, Waltham, MA, USA). Hematological tests were performed using a semi-automated hematology analyzer, the BC-2800 VET (Mindray, Shenzhen, China). The analyzer readings were calibrated for several fish species, including the grayling, based on the hematological results obtained by traditional methods [40] by its distributor, Stamar (Dąbrowa Górnicza, Poland). Plasma biochemical tests were performed using the BS-120 automated biochemical analyzer (Mindray, Shenzhen, China).

### 2.6. Statistical Analysis

The obtained values were subjected to statistical analysis in the Statistica 12 program (StatSoft, Inc., Tulsa, OK, USA). The normality of variances was tested using the Shapiro–Wilk W test. The obtained values for diploid and triploid graylings were compared using Student’s *t*-test. Differences were recorded as significant at *p* ≤ 0.05.

## 3. Results

### 3.1. Macroscopic Analysis of Gonads

**Figure 1:**
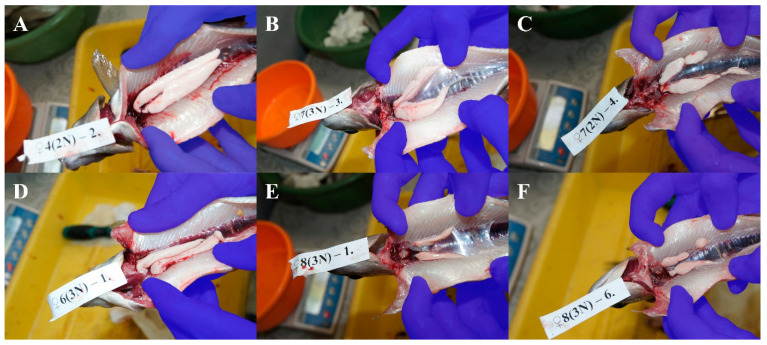
Development of gonads in diploid and triploid males: (**A**) milky white, symmetrical, spermiating; (**B**) milky white, symmetrical, non-spermiating; (**C**) milky white, asymmetrical or segmented, spermiating; (**D**) milky white, asymmetrical or segmented, non-spermiating; (**E**) pinkish, symmetrical, non-spermiating; (**F**) pinkish, asymmetrical or segmented, non-spermiating.

**Figure 2:**
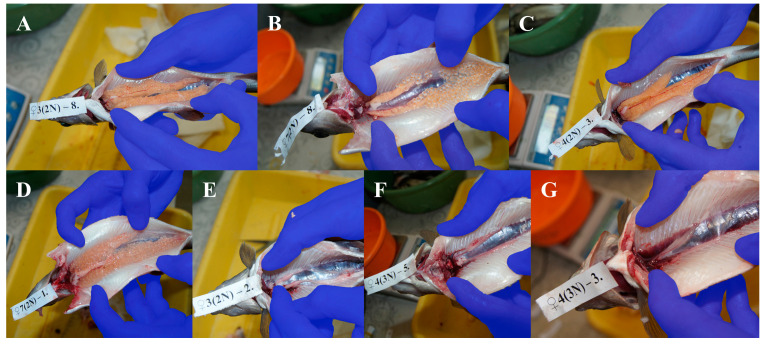
Development of gonads in diploid and triploid females: (**A**) yellow–orange, symmetrical with oocytes; (**B**) yellow–orange, symmetrical, ovulation; (**C**) yellow–orange, asymmetrical with oocytes; (**D**) yellow–orange, asymmetrical, ovulation; (**E**) pinkish milky, reduced, symmetrical, no oocytes; (**F**) pinkish transparent, reduced, symmetrical, no oocytes; (**G**) pinkish transparent, reduced, asymmetrical, no oocytes.

European graylings from the broodstock used in the present research sexually mature usually at the age of two. The examined diploid males had well-developed testes (with some asymmetry observed in less than 30% of males), and all were spermiating (Figure 1 and Table 1). In turn, triploid males were not spermiating at all, and their testes were solid, showing large variation in morphology (Table 1). The ovaries of most diploid females contained matured oocytes. Ovulated eggs were observed in about 30% of the females. Ovaries in triploid females were highly reduced and were transparent without oocytes (Figure 2 and Table 2).

### 3.2. Hematological Indices

**Figure 3:**
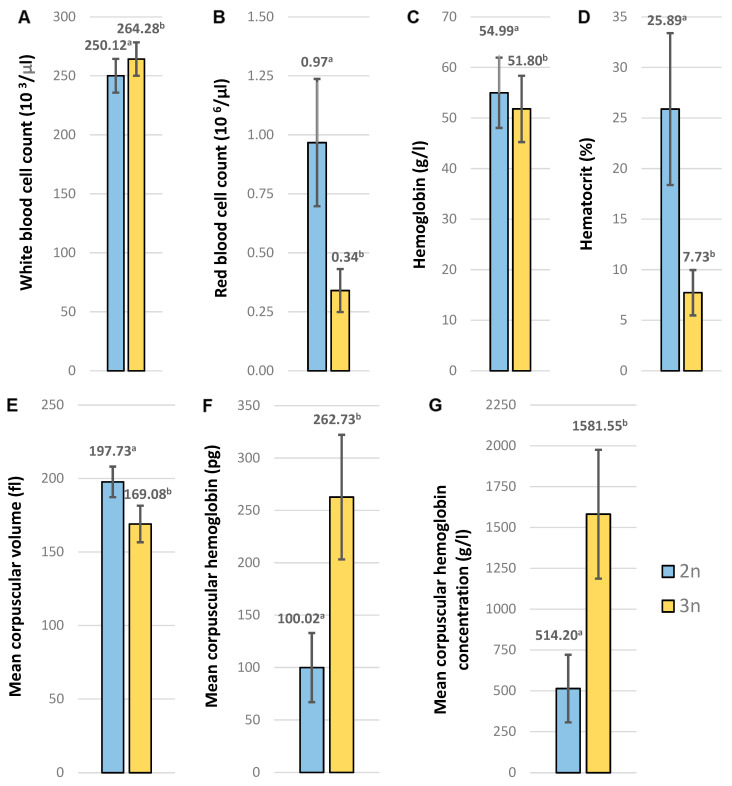
Hematological indicators of diploid (2n) and triploid (3n) European graylings: (**A**) white blood cell count; (**B**) red blood count; (**C**) hemoglobin; (**D**) hematocrit; (**E**) mean corpuscular volume; (**F**) mean corpuscular hemoglobin; (**G**) mean corpuscular hemoglobin concentration. Distinct letters indicate statistically significant (*p* ≤ 0.05) differences in the recorded values.

The hematological indices of triploid and diploid European graylings are presented in Figure 3. In triploid individuals, the white blood cell count (WBC), mean corpuscular hemoglobin (MCH) and mean corpuscular hemoglobin concentration (MCHC) were 5.66%, 162.68% and 207.57% (respectively), higher than in diploids (*p* ≤ 0.05). In turn, the red blood cell count (RBC), hemoglobin (HGB), hematocrit (HCT) and mean corpuscular volume (MCV) in triploids were 64.82%, 5.80%, 70.16% and 14.49% lower, respectively (Figure 3).

### 3.3. Biochemical Indices

Most of the biochemical indices measured in the blood plasma did not show significant differences between diploid and triploid graylings (Table 3 and Table 4); however, the concentration of triglycerides was higher by 21.96% in triploids, while the concentration of albumins was higher by 3.74% in diploids (*p* ≤ 0.05) (Table 3). Moreover, the chloride concentration was lower by 4.74% in the triploid specimens (*p* ≤ 0.05) (Table 4).

### 3.4. Erythrocyte Measurements

**Figure 4:**
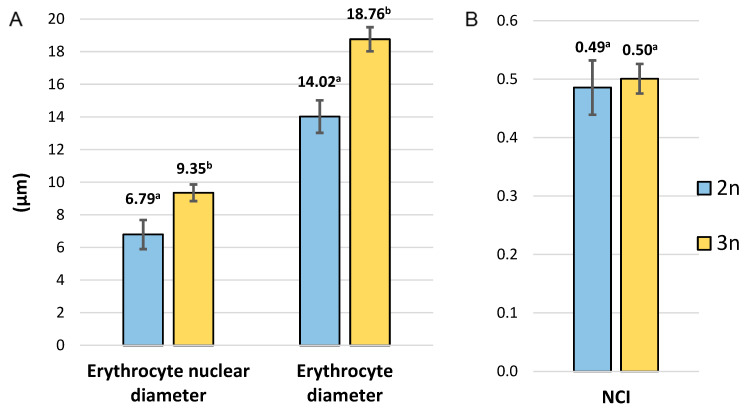
The diameters of erythrocyte nuclei and erythrocytes (**A**) and NCI (**B**) of diploid and triploid European graylings (mean (±SD)). Distinct letters indicate statistically significant (*p* ≤ 0.05) differences in the recorded values.

**Figure 5:**
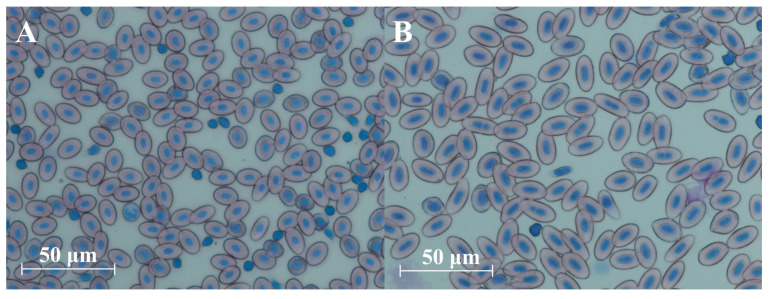
Erythrocytes of diploid (**A**) and triploid (**B**) European graylings (*Thymallus thymallus*).

The lengths of erythrocytes for diploid and triploid fish ranged from 10.99 μm to 17.97 μm and from 15.43 μm to 24.59 μm, respectively, while the lengths of the erythrocyte nuclei ranged from 4.46 μm to 9.58 μm (diploids) and from 7.32 μm to 12.59 μm (triploids). The diameters of the erythrocytes and erythrocyte nuclei of graylings from the triploid group were greater than those in fishes from the diploid group by 37.62% and 33.81%, respectively, and the differences were significant (*p* ≤ 0.05). In turn, no significant differences in the NCI between individuals from diploid and triploid groups were observed (Figure 4 and Figure 5).

## 4. Discussion

In triploids, problems with the segregation of three sets of chromosomes during meiosis result in improper gonadal development and disturbed gametogenesis, and triploid females are considered sterile [1,25,41,42]. In one-year-old triploid graylings, macroscopically visible pairs of gonads are morphologically like those of diploid specimens [34]. Only histological examination exhibited that gonadal strands in the triploid specimens were composed of connective tissues including fibroblasts, adipocytes and degenerated epithelial structures, while the gonads of their diploid counterparts contained properly formed gametes [34]. Moreover, in the case of one-year-old triploid grayling individuals, it was not possible to distinguish males and females based on macroscopic and histological analyses of their gonads. European graylings from the studied broodstock reach sexual maturity in the second year after hatching [43]; thus, it was reasonable to examine the status of gonadal development in diploids and triploids two years after hatching. The two-year-old diploid European grayling individuals analyzed in the present study were matured, with well-developed testes reported in males and ovulated oocytes observed in females (Figure 1 and Figure 2, Table 1 and Table 2). Among their triploid siblings, a simple macroscopic analysis enabled the identification of males that usually had asymmetrical and segmented testes (Figure 1) and females showing gonadal strands similar to those observed in the one-year-old triploids (Figure 2). The testes of the triploid males were well developed and, in some cases, similar to testes from the diploids. Triploid male salmonids usually develop testes with germ cells at the different stages of development/maturation [44], and in at least some of such males, spermiation has been observed [45]. In the brown trout, three-year-old and older triploids produce high-quality sperm [46]. The lack of oocytes in the ovaries of two-year-old triploid grayling females suggests that such individuals might be sterile. Nonetheless, taking into account that in triploid brown trout, gonadal differentiation is only delayed and five-year-old triploid females develop functional ovaries [46], research over a period exceeding two years needs to be performed to ascertain if triploid grayling females are sterile. 

In general, triploid individuals, when compared to diploids, show increased heterozygosity and have larger but fewer cells, and the development of their gonads is disturbed [25]. Concerning the number and size of the cells in the adult European grayling, an additional haploid set of chromosomes caused a significant increase in the diameters/dimensions of the erythrocytes and their nuclei in triploids; however, the nucleocytoplasmic index (NCI) did not differ between triploids and diploids (Figure 4). A similar pattern has also been observed in one-year-old cytogenetically verified diploid and triploid graylings [34]. The substantial differences between diploid and triploid red cell dimensions observed in the present study make erythrocyte measurement a reliable, less laborious and inexpensive approach to verifying the ploidy level in the graylings. Although it has been widely observed that the ploidy level affects cell size [25], this is not the case here, where the cell volumes (MCV) in the diploid and triploid grayling individuals do not differ significantly (Figure 3). A similar trend has been observed in triploid sturgeons, where the MCV in triploids was only slightly higher than the MCV observed in diploid specimens [47]. The relationship between the ploidy level and cell size might be much more complex than the simple assumption that increased numbers of copy genes increase the amount of proteins, which eventually results in the increased cell volume. In *Arabidopsis thaliana*, experimental data suggest that the ploidy level is not directly linked to the cell volume [48]. 

The number of erythrocytes was substantially lower in the triploid graylings when compared to diploids, which was followed by a reduction in the hematocrit (Hct), but a decreased number of red blood cells did not affect the level of total hemoglobin in the whole blood (Hb), which did not differ significantly in the specimens characterized by different ploidies (Figure 3). Although a reduced number of red cells is a common feature of triploids, the total hemoglobin level in specimens with an additional set of chromosomes showed large interspecies variation and may be comparable to that observed in diploids [49,50,51], lower [52,53,54] or higher [46]. In turn, the hemoglobin content per cell assessed in the erythrocytes (MCA) and in the volume of erythrocytes (MCHC) was substantially increased in the triploid graylings (Figure 3). A similar pattern has been observed in triploids of other species [52,55,56].

Contrary to red blood cells, no significant differences in the leucocyte counts between triploid and diploid graylings were observed. A lack of substantial differences in the white blood cells has also been described in tench [57] and Caspian trout [58]; nevertheless, other studies show decreased leucocyte counts in triploids [59]. The similar number of leucocytes in graylings of different ploidies suggests that diploid and triploid specimens have similar non-specific defense activities and comparable susceptibility to the disease.

Many studies have demonstrated that under optimal environmental conditions, the aerobic and anaerobic capacity of diploid and triploid salmonids are comparable [50,51,60,61]. However, under less favorable conditions including a higher water temperature and lower oxygen level, triploid salmonid individuals exhibit reduced performance [62,63]. Therefore, it can be inferred that triploid specimens are less resistant to the stress caused by suboptimal circumstances. In our studies, however, glucose and lactate concentrations (secondary stress markers) did not differ substantially between diploid and triploid graylings. The lack of differences in these markers may indicate that the examined fish were reared under optimal rearing conditions.

Measurements of triglyceride levels, in combination with other lipid markers, are useful in the diagnosis of hyperlipoproteinemia, dyslipidemia and triglyceridemia. Assessments of triglyceride concentrations are also used in the diagnosis and treatment of diabetes, nephrosis, liver obstruction and other diseases related to lipid metabolism or various endocrine disorders [64,65]. The levels of these parameters, along with other biochemical indicators such as total protein, albumins, globulins and ALP in blood plasma/serum, generally reflect endocrine function and the integrity of key organs, especially the liver and kidneys, and are often used to evaluate the nutritional status of fish [66,67]. In the present research, the levels of triglycerides, albumins and chloride ions differed significantly between diploids and triploids. The elevated triglyceride level observed in triploids may indicate disturbances in body lipid metabolism or even impaired liver function. In turn, the significantly reduced albumin level could be linked with malnutrition in the individuals with higher ploidy. However, the levels of the above-mentioned parameters, even if they differ between 2n and 3n, are still within the ranges that are found in healthy salmonid fish species [63], which undoubtedly suggests the proper performance of the triploid graylings.

## 5. Conclusions

An additional set of chromosomes has a large impact on gonadal development in the European grayling. Two-year-old diploid individuals have properly developed ovaries and testes filled with gametes, while triploid ovaries are highly reduced without any oocytes and triploid testes are solid and non-spermiating. Diploid females were ovulating, and males were spermiating. In turn, hematological analysis exhibited that triploids have bigger red blood cells, an elevated white blood cell count and increased mean corpuscular hemoglobin (MCH) and mean corpuscular hemoglobin concentration (MCHC) than diploids, but the red blood cell count, hemoglobin concentration (Hb) and hematocrit were higher in diploids. Smaller differences between specimens of different ploidies were observed when plasma biochemical indices were assessed. Two-year-old triploid grayling females were shown to have underdeveloped ovaries lacking oocytes; however, research over a longer period exceeding two years needs to be undertaken to state if such individuals are sterile.

## Figures and Tables

**Table 1:** Characteristics of the testes in diploid and triploid European grayling males and percentage of individuals with each particular testicle type.

Classification of Testes	Diploid Males (*n* = 25)	Triploid Males (*n* = 25)
(A) milky white, symmetrical, spermiating	72.00%	-
(B) milky white, symmetrical, non-spermiating	-	4.00%
(C) milky white, asymmetrical or segmented, spermiating	28.00%	-
(D) milky white, asymmetrical or segmented, non-spermiating	-	28.00%
(E) pinkish, symmetrical, non-spermiating	-	32.00%
(F) pinkish, asymmetrical or segmented, non-spermiating	-	36.00%

**Table 2:** Characteristics of the ovaries in diploid and triploid European grayling females and percentage of individuals with particular types of ovaries.

Classification of Ovaries	Diploid Females (*n* = 15)	Triploid Females (*n* = 15)
(A) yellow–orange, symmetrical with oocytes	33.33%	-
(B) yellow–orange, symmetrical, ovulation	26.67%	-
(C) yellow–orange, asymmetrical with oocytes	20.00%	-
(D) yellow–orange, asymmetrical, ovulation	6.67%	-
(E) pinkish milky, reduced, symmetrical, no oocytes	13.33%	-
(F) pinkish transparent, reduced, symmetrical, no oocytes	-	47.06%
(G) pinkish transparent, reduced, asymmetrical, no oocytes	-	52.94%

**Table 3:** Mean and standard deviation of plasma metabolites in diploid and triploid European graylings (*Thymallus thymallus*).

	Diploid Specimens (*n* = 40)	Triploid Specimens (*n* = 40)	Significant Differences (*p* ≤ 0.05)
Glucose (mg/dL)	79.55 ± 12.95	85.55 ± 15.93	0.068
Triglycerides (mg/dL)	298.04 ± 116.15	363.49 ± 142.53	0.027
Cholesterol (mg/dL)	515.05 ± 118.05	478.53 ± 108.00	0.153
Total protein (g/dL)	3.82 ± 0.32	3.74 ± 0.30	0.301
Albumin (g/dL)	2.06 ± 0.20	1.98 ± 0.12	0.038
Globulin (g/dL)	1.75 ± 0.21	1.76 ± 0.21	0.320
Total bilirubin (mg/dL)	0.25 ± 0.22	0.19 ± 0.18	0.191
Creatynine (mg/dL)	0.25 ± 0.09	0.25 ± 0.08	0.901
Lactate (mg/dL)	58.60 ± 22.32	68.76 ± 24.94	0.059
Ammonia (μg/dL)	534.30 ± 174.58	593.36 ± 170.37	0.130
Alanine transaminase (U/L)	51.83 ± 28.88	55.09 ± 29.95	0.622
Aspartate transaminase (U/L)	1890.07 ± 1235.95	2417.49 ± 1534.91	0.095
Alkaline phosphatase (U/L)	360.68 ± 65.26	347.40 ± 87.55	0.444
Amylase (U/L)	45.15 ± 18.32	42.50 ± 14.12	0.470

**Table 4:** Mean and standard deviation of ion concentrations in diploid and triploid European graylings (*Thymallus thymallus*).

	Diploid Specimens (*n* = 40)	Triploid Specimens (*n* = 40)	Significant Differences (*p* ≤ 0.05)
Potassium (mmol/L)	1.73 ± 0.68	1.62 ± 0.79	0.504
Sodium (mmol/L)	159.04 ± 5.60	158.34 ± 5.17	0.562
Chloride (mmol/L)	299.84 ± 29.05	285.64 ± 34.31	0.049
Phosphorus (mg/dL)	8.19 ± 1.45	8.34 ± 1.29	0.641
Calcium (mg/dL)	12.65 ± 3.19	11.76 ± 1.11	0.999
Magnesium (mg/dL)	2.73 ±0.33	2.73 ± 0.25	0.942

## Data Availability

The data in the present manuscript are available from the corresponding author upon request.

## References

[1] Piferrer F., Beaumont A., Falguière J.C., Flajšhans M., Haffray P., Colombo L. (2009). Polyploid fish and shellfish: Production, biology and applications to aquaculture for performance improvement and genetic containment. Aquaculture.

[2] Cherfas N., Gomelsky B., Ben-Dom N., Hulata G. (1995). Evidence for the heritable nature of spontaneous diploidization in common carp, *Cyprinus carpio* L., eggs. Aquac. Res..

[3] Gold J.R., Avise J.C. (1976). Spontaneous triploidy in the California roach *Hesperoleucus symmetricus* (Pisces: Cyprinidae). Cytogenet. Genome Res..

[4] Thorgaard G.H., Gall G.A. (1979). Adult triploids in a rainbow trout family. Genetics.

[5] Ocalewicz K., Dobosz S. (2009). Karyotype variation in the albino rainbow trout (*Oncorhynchus mykiss* (Walbaum)). Genome.

[6] Kendall C., Valentino S., Bodine A.B., Luer C.A. (1994). Triploidy in a nurse Shark, *Ginglymostoma cirratum*. Copeia.

[7] Zhang Q., Arai K. (1999). Aberrant meioses and viable aneuploid progeny of induced triploid loach (*Misgurnus anguillicaudatus*) when crossed to natural tetraploids. Aquaculture.

[8] Aegerter S., Jalabert B. (2004). Effects of post-ovulatory oocyte ageing and temperature on egg quality and on the occurrence of triploid fry in rainbow trout, *Oncorhynchus mykiss*. Aquaculture.

[9] Flajšhans M., Kohlmann K., Rab P. (2007). Autotriploid tench *Tinca tinca* (L.) larvae obtained by fertilization of eggs previously subjected to postovulatory ageing in vitro and in vivo. J. Fish Biol..

[10] Zhou H., Xu Q.Z., Zhang R., Zhuang Z.X., Ma Y.Q., Wang W., Ma T.Y., Sui Y., Yang L., Cao X. (2018). Gonadal transcriptome analysis of hybrid triploid loaches (*Misgurnus anguillicaudatus*) and their diploid and tetraploid parents. PLoS ONE.

[11] Janko K., Bohlen J., Lamatsch D., Flajšhans M., Epplen J.T., Ráb P., Kotlík P., Šlechtová V. (2007). The gynogenetic reproduction of diploid and triploid hybrid spined loaches (Cobitis: Teleostei), and their ability to establish successful clonal lineages—On the evolution of polyploidy in asexual vertebrates. Genetica.

[12] Juchno D., Boroń A. (2006). Age, reproduction and fecundity of the spined loach *Cobitis taenia* L. (Pisces, Cobitidae) from Lake Klawój (Poland). Reprod. Biol..

[13] Xiao J., Zou T., Chen Y., Chen L., Liu S., Tao M., Zhang C., Zhao R., Zhou Y., Long Y. (2011). Coexistence of diploid, triploid and tetraploid crucian carp (*Carassius auratus*) in natural waters. BMC Genet..

[14] Alves M.J., Gromicho M., Collares-Pereira M.J., Crespo-López E., Coelho M.M. (2004). Simultaneous production of triploid and haploid eggs by triploid *Squalius alburnoides* (Teleostei: Cyprinidae). J. Exp. Zoolog. Part A Comp. Exp. Biol..

[15] Morishima K., Oshima K., Horie S., Fujimoto T., Yamaha E., Arai K. (2004). Clonal diploid sperm of the diploid-triploid mosaic loach, *Misgurnus anguillicaudatus* (Teleostei: Cobitidae). J. Exp. Zoolog. Part A Comp. Exp. Biol..

[16] Chourrout D., Chevassus B., Krieg F., Happe A., Burger G., Renard P. (1986). Production of second generation triploid and tetraploid rainbow trout by mating tetraploid males and diploid females—Potential of tetraploid fish. Theor. App. Genet..

[17] Blanc J.M., Chourrout D., Krieg F. (1987). Evaluation of juvenile rainbow trout survival and growth in half-sib families from diploid and tetraploid sires. Aquaculture.

[18] Quillet E., Chevassus B., Devaux A. (1986). Timing and duration of hatching in gynogenetic, triploid, tetraploid, and hybrid progenies in rainbow trout. Genet. Sel. Evol..

[19] Myers J.M., Hershberger W.K. (1991). Early growth and survival of heat-shocked and tetraploid-derived triploid rainbow trout (*Oncorhynchus mykiss*). Aquaculture.

[20] Arai K., Fujimoto T. (2013). Genomic constitution and atypical reproduction in polyploid and unisexual lineages of the *Misgurnus loach*, a teleost fish. Cytogenet. Genome. Res..

[21] Ueda T., Kobayashi M., Sato R. (1986). Triploid rainbow trouts induced by polyethylene glycol. Proc. Japan Acad. Ser. B.

[22] Grunina A.S., Recoubratsky A.V., Tsvetkova L.I., Barmintsev V.A. (2006). Investigation on dispermic androgenesis in sturgeon fish. The first successful production of androgenetic sturgeons with cryopreserved sperm. Int. J. Refrig..

[23] Thorgaard G.H. (1986). Ploidy manipulation and performance. Aquaculture.

[24] Pandian T.A., Koteeswaran R. (1998). Ploidy induction and sex control in fish. Hydrobiologia.

[25] Benfey T.J. (1999). The physiology and behavior of triploid fishes. Rev. Fish. Sci..

[26] Arai K. (2001). Genetic improvement of aquaculture finfish species by chromosome manipulation techniques in Japan. Aquaculture.

[27] Northcote T.G. (1995). Comparative biology and management of Arctic and European grayling (Salmonidae, *Thymallus*). Rev. Fish. Biol. Fish..

[28] Persat H., Kirchhofer A., Hefti D. (1996). Threatened populations and conservation of the European *T. thymallus*, *Thymallus thymallus* (L. 1758). Conservation of Endangered Fishes of Europe.

[29] Koskinen M.T., Primmer C.R. (2001). Interpopulation genetic divergence in European grayling (*Thymallus thymallus*, Salmonidae) at a microgeographic scale: Implications for conservation. Conserv. Genet..

[30] Susnik S., Berrebi P., Dovcˇ P., Hansen M.M., Snoj A. (2004). Genetic introgression between wild and stocked salmonids and the prospects for using molecular markers in population rehabilitation: The case of the Adriatic grayling (*Thymallus thymallus* L. 1785). Heredity.

[31] Weiss S.J., Kopun T., Sušnik Bajec S. (2013). Assessing natural and disturbed population structure in European grayling *Thymallus thymallus*: Melding phylogeographic, population genetic and jurisdictional perspectives for conservation planning. J. Fish. Biol..

[32] Koskinen M.T., Piironen J., Primmer C.R. (2002). Genetic assessment of spatiotemporal evolutionary relationships and stocking effects in grayling (*Thymallus thymallus*, Salmonidae). Ecol. Lett..

[33] Duftner N., Koblmuller S., Weiss S., Medgyesy N., Sturmbauer C. (2005). The impact of stocking on the genetic structure of European grayling (*Thymallus thymallus*, Salmonidae) in two alpine rivers. Hydrobiologia.

[34] Hliwa P., Panasiak L., Ziomek E., Rożyński R., Leonowicz Ł., Grudniewska J., Dobosz S., Ocalewicz K. (2022). Application of high hydrostatic pressure (HHP) shock to induce triploid development in the European grayling (*Thymallus thymallus* L.). Anim. Reprod. Sci..

[35] Oliveira A.T., Lemos J.R.G., Santos M.Q.C., Pantoja-Lima J., Aride P.H.R., Araújo M.L.G., Tavares-Dias M., Marcon J.L. (2021). Morphological, cytochemical and ultrastructural aspects of blood cells in freshwater stingray species in the middle Rio Negro basin of Amazonian. Brazil. Sci. Rep..

[36] Witeska M., Kondera E., Lugowska K., Bojarski B. (2022). Hematological methods in fish—Not only for beginners. Aquaculture.

[37] Santos M.Q.D., Aride P.H.R., Farias F.D.F., Oliveira A.T. (2024). Hematological and plasma biochemical profile of two species of freshwater stingrays from the Amazon. Vet. Res. Commun..

[38] Rożyński R., Kuciński M., Dobosz S., Ocalewicz K. (2023). Successful application of UV-irradiated rainbow trout (*Oncorhynchus mykiss*) spermatozoa to induce gynogenetic development of the European grayling (*Thymallus thymallus*). Aquaculture.

[39] Zakęś Z., Demska-Zakęś K., Rożyński M., Gomułka P., Rożyński R. (2022). Influence of intraperitoneal implantation of 12 mm PIT on the welfare of juvenile brown trout (*Salmo trutta*). Fisheries Research.

[40] Dacie J.V., Lewis S.M., Lewis S.M., Bain B.J., Bates I. (2001). Practical Haematology. Practical Heamatology.

[41] Lincoln R.F., Scott A.P. (1983). Production of all-female triploid rainbow trout. Aquaculture.

[42] Benfey T.J. (2001). Use of sterile triploid Atlantic salmon (*Salmo salar* L.) for aquaculture in New Brunswick, Canada. ICES J. Mar. Sci..

[43] Szmyt M., Dobosz S., Kucharczyk D., Grudniewska J., Lejk A.M. (2013). Impact of selected hormonal agents on the effectiveness of controlled reproduction of cultivated female European grayling. Fish. Aquat. Life.

[44] Carrasco L.A., Doroshov S., Penman D.J., Bromage N. (1998). Long term, quantitative analysis of gametogenesis in autotriploid rainbow trout, *Oncorhynchus mykiss*. J. Reprod. Fert..

[45] Lahnsteiner F., Lahnsteiner E., Kletzl M. (2020). Age and species related differences in gonad development of triploid Salmonidae. J. Appl. Aquac..

[46] Lahnsteiner F., Duenser A. (2024). Triploid brown trout, *Salmo trutta*, develop functional gonads with age and are able to interbreed with diploid counterparts. J. Fish Biol..

[47] Rożyński M., Demska-Zakęś K., Fopp-Bayat D. (2015). Hematological and blood gas profiles of triploid Siberian sturgeon (*Acipenser baerii Brandt*). Arch. Pol. Fish..

[48] Tsukaya H. (2013). Does Ploidy Level Directly Control Cell Size? Counterevidence from Arabidopsis Genetics. PLoS ONE.

[49] Aliah R.S., Inada Y., Yamaoka K., Taniguchi N. (1991). Effects of triploidy on haematological characterstics and oxygen consumption of ayu. Nippon. Suisan Gakkaishi.

[50] Stillwell E.J., Benfey T.J. Haemoglobin level, metabolic rate and swimming performance in triploid brook trout (*Salvelinus fontinalis*). Proceedings of the High Performance Fish.

[51] Stillwell E.J., Benfey T.J. (1996). Hemoglobin level, metabolic rate, opercular abduction rate and swimming efficiency in female triploid brook trout (*Salvelinus fontinalis*). Fish Physiol. Biochem..

[52] Graham M.S., Fletcher G.L., Benfey T.J. (1985). Effect of triploidy on blood oxygen content of Atlantic salmon. Aquaculture.

[53] Small S.A., Randall D.J. (1989). Effects of triploidy on the swimming performance of coho salmon (*Oncorhynchus kisutch*). Can. J. Fish. Aquat. Sci..

[54] Yamamoto A., Iida T. (1994). Haematological characters tics of triploid rainbow trout. Fish Pathol..

[55] Benfey T.J., Sutterlin A.M. (1984). The haematology of triploid and landlocked Atlantic salmon, *Salmo salar* L.. J. Fish Biol..

[56] Parsons G.R. (1993). Comparisons of triploid and diploid white crappies. Trans. Am. Fish. Soc..

[57] Svobodová Z., Kolářová J., Flajšhans M. (1998). The first findings of the differences in complete blood count between diploid and triploid tench, *Tinca tinca* L.. Acta Vet. Brno.

[58] Dorafshan S., Kalbassi M.R., Pourkazemi M., Mojazi A.B., Karimi S. (2008). Effects of triploidy on the Caspian salmon Salmo caspius haematology. Fish Physiol. Biochem..

[59] Benfey T.J., Biron M. (2000). Acute Stress Response in Triploid Rainbow Trout (*Oncorhynchus mykiss*) and Brook Trout (*Salvelinus fontinalis*). Aquaculture.

[60] Stillwell E.J., Benfey T.J. (1997). The critical swimming velocity of diploid and triploid brook trout. J. Fish Biol..

[61] Hyndman C.A., Keiffer J.D., Benfey T.J. (2003). The physiological response of diploid and triploid brook trout to exhaustive exercise. Comp. Biochem. Physiol. A.

[62] Altimiras J., Axelsson M., Claireaux G., Lefrancois C., Mercier C., Farrell A.P. (2002). Cardiorespiratory status of triploid brown trout during swimming at two acclimation temperatures. J. Fish Biol..

[63] Hyndman C.A., Kieffer J.D., Benfey T.J. (2003). Physiology and survival of triploid brook trout following exhaustive exercise in warm water. Aquaculture.

[64] Rifai N., Bachorik P.S., Albers J.J., Burtis C., Ashwood E.R. (2001). Lipids, Lipoproteins, and Apolipoproteins in Tietz Fundamentals of Clinical Chemistry.

[65] Amal S., Mohamed M.A.E., Desoky Nahed S.G. (2019). The Changes in Triglyceride and Total Cholesterol Concentrations in the Liver and Muscle of Two Fish Species from Qarun Lake, Egypt. Oceanogr. Fish Open Access J..

[66] Folmar L.C. (1993). Effects of chemical contaminants on blood biochemistry of teleost fish: A bibliography and synopsis of selected effects. Envir. Toxicol. Chem..

[67] Zubay G. (1993). Biochemistry.

